# Tumour karyotype discriminates between good and bad prognostic outcome in neuroblastoma.

**DOI:** 10.1038/bjc.1988.24

**Published:** 1988-01

**Authors:** H. Christiansen, F. Lampert

**Affiliations:** Kinderpoliklinik, Justus-Liebig-University, Giessen, West Germany.

## Abstract

**Images:**


Br. J. Cancer (1988), 57, 121–126 © The Macmillan Press Ltd., 1988

### Tumour karyotype discriminates between good and bad prognostic outcome in neuroblastoma

H. Christiansen & F. Lampert

*Kinderpoliklinik, Justus-Liebig-University, D-6300 Giessen, West Germany.*

**Summary** In 28 patients with neuroblastoma of different stages the karyotype was determined in the primary tumour and/or in the metastases by direct chromosome preparation or short term cell culture. In addition, DNA analysis for the proto-oncogene N-*myc* was performed for comparison in 10 cases.

Abnormalities (deletions, translocations, derivations) of the short arm of chromosome 1 with the most frequent breakpoint at 1p32 (besides rarer aberrations in other chromosomes) were found in the tumour karyotype of 15 of 18 (83%) patients with metastatic disease (stage IV) and in 2 of 3 patients with stage III, but in none of the 7 patients with stages I, II, IV-S who are all alive with no evidence of disease. These 7 surviving patients with good prognosis had a hyperploid tumour karyotype, mainly in the triploid range. Eleven of the 18 (61%) patients with stage IV and 1 of 3 patients with stage III also contained double minutes (DMs) and/or homogeneously staining regions (HSRs) in their tumour karyotypes. N-*myc* amplification (30 to 60 copies) in the tumour DNA was detected in 2 of 6 (33%) examined cases with stage IV, in 1 out of 2 examined cases with stage III, and correlated with the presence of DMs/HSRs.

Life table analysis showed a 90% probability of surviving in patients lacking the 1p abnormality as compared to less than 10% in patients with an aberrant 1p chromosome in the tumour cells. We conclude that tumour karyotype, in particular the structure of the short arm of chromosome 1, is the most important factor in determining the different outcome in children with neuroblastoma.

After brain tumours neuroblastoma represents the most common solid tumour in childhood with an annual incidence of 8 cases per million children under 15 years; most of them – in contrast to the more frequent childhood leukaemias – occurring within the first 3 years of life.

The biological behaviour of neuroblastoma is unique and still puzzling to paediatric oncologists in that there is spontaneous regression in congenital tumours (even in the metastatic stage) and an almost 100% survival rate in localized tumours as compared to a less than 10% survival chance in non-localized, disseminated tumours in children over 1 year of age in spite of intensive chemotherapy (Berthold *et al.*, 1986). Besides known prognostic factors such as age and tumour stage additional clinical factors useful in predicting outcome, such as serum ferritin and histologic type, have recently been emphasized (Evans *et al.*, 1987). Also, the DNA properties of neuroblastoma cells such as the increased copy number of the proto-oncogene N-*myc* (Brodeur *et al.*, 1984; Seeger *et al.*, 1985) or the increased expression of the oncogene (Rosen *et al.*, 1986) were found to be associated with progressive growth of the tumour.

That tumour karyotype may be important in the prognosis of human neuroblastoma was proposed by us after we obtained tumour cytogenetic data in a series of 14 children with neuroblastoma (Franke *et al.*, 1986*a*). In an extended series of 28 patients with all the different tumour stages we now confirm the presence or absence of the chromosome 1 short arm abnormality as the most decisive factor characterizing the different biological behaviour of neuroblastoma cell clones and thus the clinical outcome.

### Materials and methods

Patients were registered and prospectively treated within the consecutive Neuroblastoma Trials NB 79, NB 82, and NB 85 of the German Paediatric Oncology Group which allowed uniform treatment, documentation, pathological evaluation, and continuous control of all clinical parameters (Berthold *et al.*, 1986).

Tumour tissue samples were collected at operation or sent in by express mail, and were taken either prior to or at least 2 months after chemotherapy. Samples were immediately transferred to short-term cultures for 3 h, and/or 2, 3 and at most up to 8 days. Methods pertaining to chromosome preparation from solid tumours have been described previously (Franke *et al.*, 1986*b*). Chromosome preparations of bone marrow metastases were performed after cell synchronisation with methotrexate. Chromosomes were G-banded with 3% Giemsa solution (pH 6.8) after pretreatment with trypsin. The best banded metaphases were photographed and karyotyped. In the majority of the patients more than 20 tumour metaphases could be analyzed.

DNA analysis of the N-*myc* gene from the same tumour cell material that was used for chromosome study could be performed in 10 patients. Isolated DNA was digested with EcoRI, electrophoresed, and transferred on to filter membranes (Du Pont). N-*myc* amplification was tested with the blotting method (Southern) using the probe NB-1 (Schwab *et al.*, 1983) which detects 2.0 Kb EcoRI fragments. The extent of N-*myc* amplification was confirmed by dot-blot analyses via serial dilution of the tumour-DNA.

### Results

Chromosomes could be successfully prepared and analyzed from neuroblastoma tissue in 28 patients out of a total of about 90 patients examined.

The clinical and the most important cytogenetic data such as the 1p abnormality, DMs and HSRs, including N-*myc* copies in the tumour where DNA analysis was performed, are summarized in Table I. In Table II the complete tumour karyotypes, including the number of cells counted and analyzed are listed. In the bone marrow preparations diploid cells of normal haematopoiesis were also found and could be excluded. The tumour cell preparations showed cell populations of clonal origin. In only a few cases 1 to 3 subpopulations with minor cytogenetic variations were seen (Table II).

Of the 12 infants studied, 4 of the 6 with stage IV disease are dead; the others, including 3 with stage IV-S, 2 with stage II and 1 with stage I tumours, are all alive. All 4 dead infants had a chromosome 1p abnormality in the tumour karyotype, and DMs/HSRs in 3. Of the 2 living infants, however, with stage IV none was without structural tumour chromosome aberrations. Patient Mo-K had a 6q-deletion

Correspondence: F. Lampert.
Received 28 July 1987; and in revised form 23 November 1987.

**Table I** Major clinical, tumour-cytogenetic and tumour-DNA findings in 28 patients with neuroblastoma

| *Patient* | *Age at diagnosis (mo)* | *Sex* | *Survival (mo)*[a] | *Outcome* | *Stage* | *Tissue* | *Modal number (range)* | *Chromosome-aberrations* No. 1 | *dms* | *hsr* | *N*-myc *copies*[b] |
|---|---|---|---|---|---|---|---|---|---|---|---|
| JC-H | 1 | m | 35 | alive, NED | I | tu | 58 (54–64) | – | – | – | nt |
| RZ-GI | 73 | f | 44 | alive, NED | II | tu | (39–78) | – | – | – | nt |
| TT-DO | 11 | m | 53 | alive, NED | II | tu | 70 (45–71) | – | – | – | nt |
| MJ-GI | pp | m | 10 | alive, NED | II | tu | (82–84) | – | – | – | 1 |
| JS-DO | 2 | f | 19 | alive, NED | IVs | tu | (55–60) | – | – | – | 1 |
| SB-S | 1 | f | 33 | alive, NED | IVs | tu | (64–68) | – | – | – | nt |
| JK-NE | 3 | m | 51 | alive, NED | IVs | tu | (56–58) | – | – | – | nt |
| MB-MR | 54 | m | 75 | alive | III | tu | 60 (54–71) | – | – | – | 1 |
| MB-H | 17 | f | 3 | dead | III | tu | 47 (46–47) | + | – | + | 30 |
| FW-KS | 43 | m | 29 | alive, NED | III | tu | (46–64) | + | – | – | nt |
| MO-K | 9 | f | 25 | alive | IV | bm | (31–88) | – | – | – | 1 |
| JF-GÖ | 35 | f | 8 | dead | IV | bm | 46 (45–46)/ | – | – | – | 1 |
| | | | | | | | 64 (63–66) | – | – | – | 1 |
| RB-GI | 45 | m | 23 | dead | IV | bm | 46 (43–46) | – | – | – | 1 |
| ME-HER | 23 | m | 5 | dead | IV | bm | (30–83) | + | – | – | 1 |
| MB-N | 119 | m | 1 | dead | IV | tu | 57 (43–62) | + | – | – | nt |
| WW-BT | 17 | m | 14 | dead | IV | bm | (80–108) | + | – | – | nt |
| SS-HD | 11 | m | 6 | dead | IV | bm | 46 (26–46) | + | – | – | nt |
| SC-H | 10 | m | 3 | alive | IV | tu | 73 (48–75) | + | + | – | nt |
| FO-ER | 15 | m | 7 | alive | IV | bm | 46 (43–47) | + | + | – | 30 |
| CR-GI | 8 | f | 5 | dead | IV | tu | 46 (44–47) | + | + | – | nt |
| | | | | | | bm | 46 (45–46) | + | + | – | nt |
| VS-GI | 15 | f | 7 | dead | IV | bm | 46 (44–48) | + | + | – | nt |
| SB-GI | 26 | m | 16 | dead | IV | tu | 84 (82–90) | + | + | – | nt |
| | | | | | | bm | 101 (99–103) | + | + | – | nt |
| AG-GI | 30 | f | 17 | dead | IV | bm | (92–174) | + | + | – | nt |
| | | | | | | bm-rel | (46–95) | + | + | – | nt |
| MG-N | 9 | f | 16 | dead | IV | bm | 45 (38–48) | + | + | – | nt |
| | | | | | | bm-rel | 46 (42–48)/ | + | + | + | 60 |
| | | | | | | | 46 (43–47)/ | + | + | + | 60 |
| | | | | | | | 46 (44–46) | + | – | + | 60 |
| MH-NOH | 43 | f | 32 | dead | III>IV | ln | 61 (44–73) | + | + | + | nt |
| SW-DO | 35 | f | 13 | dead | III>IV | bm | 46 (42–47) | + | – | + | nt |
| MK-WÜ | 63 | f | 16 | dead | III>IV | tu | (34–88) | + | – | + | nt |
| SJ-B | 9 | m | 4 | dead | III>IV | bm | (63–84) | + | – | + | nt |

[a]Survival, as of 1 July 1987; [b]Single copy/haploid genome = 1. nt: not tested, pp: post partum, NED: no evidence of disease, tu: tumour, bm: bone marrow, ln: lymphnode, rel: relapse.

but no 1p abnormality, but patient SC-H had a 1p translocation (Figure 1), and thus, prognosis in this case could be bad in regard to the short observation time. All the other infants are alive with no evidence of disease. None of them had a 1p abnormality (Figure 2) and/or DMs/HSRs in the tumour karyotype, but all had hyperploid modal chromosome numbers of 58, 70, (82–84), (55–60), (64–68), (56–58), (31–88), i.e. mainly in the triploid range.

In contrast, 13 of the 15 (87%), mainly older, patients with stage IV neuroblastoma who already died, had an 1p aberrant karyotype, and 9 (60%) of them showed DMs and/or HSRs. Modal chromosome numbers were in the diploid range in about half of the patients. In the last 4 patients of the table, initially (probably falsely?) diagnosed as stage III, tumour karyotyping was done later when metastases were prominent.

As to morphology of the tumour chromosomes we purposely selected for demonstration a complete and partial karyotype from hyperploid primary tumours of infancy (Figures 1 and 2) as these usually are more difficult to prepare and analyze as compared to pseudodiploid tumour cells from bone marrrow metastases.

In the 3 cases where 30–60 copies of the N-*myc* gene were detected in the genome there were also DMs and/or HSRs in the tumour karyotype. Lack of 1p abnormality was also associated with lack of N-*myc* amplification except for one (already dead) patient (ME-HER) with stage IV and a derivated chromosome 1. DMs and HSRs never occurred simultaneously in the same metaphase cell but were encountered in different metaphases of the same cell population. HSRs were detected at different chromosomes and on different arms, e.g. at 4p, 6q, 9q, 11q, 15p, 19q, 20q. The only 2 non-surviving patients (JF-GÖ, RB-GI) with stage IV and no 1p abnormality nor DMs/HSRs or N-*myc* amplification did show, however, other structural aberrations and pseudodiploid tumour karyotype.

Overall, structural aberrations were found in the tumour karyotypes of 23 patients (18 with stage IV, 3 with stage III, 2 with stage II) – whereas in 5 patients (3 with stage IV-S, 1 with stage II, 1 with stage I) only numerical aberrations were present. As listed in Table II structural aberrations of the tumour karyotype mainly affected the short arm of chromosome 1. Other chromosomes not infrequently involved were nos. 6, 11, 12, 17 (long arm).

As to localization of the breakpoint in the abnormal 1p tumour chromosome, 12 patients could be precisely analyzed, and 1p32 was found to be the most frequently involved (Figure 3). The correlation of karyotypic pattern – taking the 1p abnormality as the most characteristic chromosome aberration – with survival could be demonstrated in 26 patients by life table analysis (Kaplan-Meier method, kindly carried out by Dr P. Kaatsch, Mainz) (Figure 4). The two most recent patients SC-H, FO-ER with stage IV were excluded because of insufficient observation time. The clear-cut distinction between two groups of patients with neuroblastoma is obvious: Patients ($n = 11$)

**Table II** Tumour cell karyotypes in 28 patients with neuroblastoma

| *Patient* | *Tissue* | *Number of metaphases counted* | *Number of metaphases analyzed* | *Modal chromosome number (range)* | *1p aberrations* | *Metaphases with additional structural aberrations* | | |
|---|---|---|---|---|---|---|---|---|
| | | | | | | *1p aberrations: absol.* | *perc.* | |
| 1. JC-H | tu | 20 | 9 | 58 (54–64) | – | 0/9 | (–) | – |
| 2. RZ-GI | tu | 61 | 16 | (39–78) | – | 0/16 | (–) | t (7;21)(p22;p13), t(19;?)(p13.1;?) |
| 3. TT-DO | tu | 40 | 15 | 70 (45–71) | – | 0/15 | (–) | t(19;?)(p13;?) |
| 4. MJ-GI | tu | 6 | 3 | (82–84) | – | 0/3 | (–) | – |
| 5. JS-DO | tu | 5 | 3 | (55–60) | – | 0/3 | (–) | – |
| 6. SB-S | tu | 3 | 2 | (64–68) | – | 0/2 | (–) | – |
| 7. JK-NE | tu | 3 | 2 | (56–58) | – | 0/2 | (–) | – |
| 8. MB-MR | tu | 37 | 11 | 60 (54–71) | – | 0/11 | (–) | der(1q), der(5p), t(6;?)(q25;?), 2 mar |
| 9. MB-H | tu | 42 | 8 | 47 (46–47) | der(1p) | 2/8 | (25%) | hsr(9)(q22), t(12;17)(q14;q21), t(16;?)(p13;?) |
| 10. FW-KS | tu | 3 | 3 | (46–64) | t(1;?)(p36;?) | 3/3 | (100%) | dup(12)(q13q24), 2 mar |
| 11. MO-K | bm | 11 | 11 | (31–88) | – | 0/11 | (–) | del(6)(q23) |
| 12. JF-GÖ | bm | 27 | 9 | 46 (45–46)/ | – | 0/9 | (–) | del(11)(q13q21) |
| | | | 6 | 64 (63–66) | – | 0/6 | (–) | del(11)(q13q21), i(12q), t(13;?)(p11;?), 1 mar |
| 13. RB-GI | bm | 54 | 17 | 46 (43–46) | – | 0/17 | (–) | del(3)(p21), del(11)(p11), 1 mar |
| 14. ME-HER | bm | 15 | 9 | (30–83) | der(1p) | 9/9 | (100%) | 6 mar |
| 15. MB-N | tu | 84 | 15 | 57 (43–62) | del(1)(p32) | 15/15 | (100%) | t(13;?)(q34;?), 4 mar |
| 16. WW-BT | bm | 21 | 11 | (80–108) | der(1p) | 7/11 | (64%) | – |
| 17. SS-HD | bm | 32 | 16 | 46 (26–46) | t(1;?)(p22;?) | 14/16 | (88%) | der(3q), der(9p), der(11q), der(12p), der(18q), 1 mar |
| 18. SC-H | tu | 7 | 5 | 73 (48–75) | t(1;?)(p22;?) | 5/5 | (100%) | 1 mar, dms |
| 19. FO-ER | bm | 17 | 15 | 46 (43–47) | del(1)(p22) | 6/15 | (40%) | dms |
| 20. CR-GI | tu | 178 | 17 | 46 (44–47) | t(1;11)(p32;p15) | 17/17 | (100%) | del(2)(p21), der(7p), dms |
| | bm | 99 | 31 | 46 (45–46) | t(1;11)(p32;p15) | 31/31 | (100%) | del(2)(p21), der(7p), dms |
| 21. VS-GI | bm | 60 | 17 | 46 (44–48) | t(1;1)(p32;q21) | 15/17 | (88%) | inv(2)(p13p23), t(8;?)(p;?), dms |
| 22. SB-GI | tu | 477 | 31 | 84 (82–90) | t(1;13;?)(p32;q34;?) | 31/31 | (100%) | inv(5)(p15;q11)del(q13.2), dms |
| | bm | 70 | 18 | 101 (99–103) | t(1;13;?)(p32;q34;?) | 18/18 | (100%) | inv(5)(p15;q11)del(q13.2), dms |
| 23. AG-GI | bm | 43 | 32 | (92–174) | del(1)(p3?) | 30/32 | (94%) | der(11p), 3 mar, dms |
| | bm-rel | 16 | 10 | (46–95) | del(1)(p3?) | 10/10 | (100%) | der(11p), dms |
| 24. MG-N | bm | 21 | 11 | 45 (38–48) | t(1;?)(p22;?) | 11/11 | (100%) | t(2;?)(p24;?), dms |
| | bm-rel | 71 | 13 | 46 (42–48)/ | t(1;?)(p22;?) | 13/13 | (100%) | t(2;?)(p24;?), t(4;6)(q31;q25), hsr(15p), der(17q), dms |
| | | | 34 | 46 (43–47)/ | t(1;?)(p22;?) | 34/34 | (100%) | t(2;?)(p24;?), t(4;6)(q31;q25), hsr(15p), der(17q), hsr(19q), dms |
| | | | 16 | 46 (44–46) | t(1;?)(p22;?) | 16/16 | (100%) | t(2;?)(p24;?), t(4;6)(q31;q25), hsr(15p), der(17q), hsr(19q) |
| 25. MH-NOH | ln | 80 | 21 | 61 (44–73) | dic(1)(p13) | 20/21 | (95%) | hsr(6)(q21), hsr(11)(q25), t(6;X)(q21;q13), i(17q), 1 mar, dms |
| 26. SW-DO | bm | 43 | 17 | 46 (42–47) | t(1;2)(p21;p36) | 17/17 | (100%) | t(2;?)(p23;?), hsr(4)(p16), t(6;?)(q15;?), t(10;?)(q24;?), hsr(11)(q13), i(17q), der(20p) |
| 27. MK-WÜ | tu | 28 | 12 | (34–88) | der(1p) | 12/12 | (100%) | 2 mar (hsr) |
| 28. SJ-B | bm | 22 | 11 | (63–84) | t(1;?)(p32;?) | 11/11 | (100%) | t(5;11)(q13;q23)del(5)(q11q13), del(10)(p11), del(12)(q13), hsr(20q) |

tu: tumour, bm: bone marrow, ln: lymph node, rel: relapse.

with a normal set of chromosome 1 in the tumour had a probability of survival of 90% *versus* the patients ($n = 15$) with an aberrant chromosome 1 having less than 10% survival probability. A comparison of 1p-aberration *versus* DMs/HSRs in the different stages of neuroblastomas shows a more pronounced stage correlation of the chromosome 1p aberration (Figure 5).

### Discussion

We succeeded in determining the tumour karyotype of 7 children with localized neuroblastoma stages I, II and stage IV-S, the special congenital form of disseminated neuroblastoma. These patients are alive and have a good prognosis. These cases were characterized oncocytogenetically by hyperploidy in the triploid range, lack of chromosome 1p abnormality and lack of cytogenetic phenomena of gene amplification such as DMs and/or HSRs (also confirmed by the absence of N-*myc* amplification). Our observations are in accordance with recent data of Kaneko *et al.* (1987) who found a near-triploid tumour lacking 1p abnormality and/or DMs, HSRs in 7 children with stage I or II who were alive with no evidence of disease, and also with data of 6 Japanese infants with stage I, II, III neuroblastoma found by mass screening for vanillylmandelic acid in the urine who only had hyperploid tumours with modal chromosome numbers ranging from 67 to 71 as their sole abnormality (Hayashi *et al.*, 1986). By measuring the DNA content of the tumour cells in 35 infants with neuroblastoma (Look *et al.*, 1984) it was also reported that hyperdiploidy was associated with a better response to chemotherapy compared to diploid tumours and that infants ($n = 4$) with IV-S stage had hyperdiploid tumour cells of clonal origin. Another investigation by flow cytometric DNA analysis in 38 cases confirmed the favorable outcome in neuroblastoma patients having an aneuploid tumour stem-line (Gansler *et al.*, 1986).

We would like to emphasize that karyotyping of the tumour cell clone by analysis of individual chromosomes has greater potential in discriminating two distinct prognostic groups in children with neuroblastoma as compared to cytophotometry on one hand, and N-*myc* determination on the other. In particular, this can be shown in poor prognosis patients, i.e. with stage IV disease: 83% (15 of 18) of our patients with tumour dissemination, being either over or

**Figure 1** Complete karyotype of a hyperploid (mn = 73) cell from the primary tumour of a 10 month old infant with neuroblastoma stage IV. Note the abnormal short arms of 2 chromosomes 1 with deletion and (unknown) translocation (breakpoint at 1p22), the presence of several DMs and one unidentified marker.

**Figure 2** Partial karyotype showing 4 normal chromosomes 1 (a) taken from a hyperploid (mn = 82) cell; (b) of the primary tumour of a newborn with neuroblastoma stage II.

**Figure 3** Localization of breakpoints – where precise analysis was possible – in the abnormal 1p chromosome of the tumour karyotype of 12 patients with neuroblastoma.

**Figure 4** Life table analysis (Kaplan-Meier method) of 26 patients with neuroblastoma of different stages with or without chromosome 1p abnormality in the tumour karyotype.

**Figure 5** Distribution of the chromosome 1p-aberration *versus* DMs/HSRs in the tumour karyotype of 28 patients with neuroblastoma of different clinical stages.

under one year, expressed a chromosome 1p abnormality in the tumour karyotype, with a diploid karyotype in only half of the patients, 61% also had DMs and/or HSRs, but only 33% (2 of 6) of the few examined cases had N-*myc* amplification. In the 13 patients with stage IV examined by Kaneko *et al.* (1987) 9 (69%) had a 1p abnormality, and 8 (62%) also had DMs/HSRs. Brodeur *et al.* (1984) found N-*myc* amplification (more than 3 copies) in neuroblastoma tissue from 13 of 25 (52%) untreated patients with stage IV, and Seeger *et al.* (1985) in 19 of 40 (48%) patients with stage IV. As one could expect a close correlation between N-*myc* amplification and presence of DMs/HSRs in the karyotype, as has also proven in one of our patients (MG-N) by *in-situ* hybridization (Christiansen *et al.*, 1987), multiple copies of N-*myc* would be present in about 60% of patients with stage IV. As a chromosome 1p abnormality is encountered more frequently in this stage of progressive tumour growth one might speculate that the aberration of chromosome 1 distal to band 1p22 (Brodeur *et al.*, 1981; Gilbert *et al.*, 1984) or 1p13 (our results) is the primary event, and gene amplification is secondary, being rarely associated with morphologic uniformity. As shown by our analysis, HSRs were distributed among several chromosomes and different arms, involving the short arm of chromosomes 4 and 15 and the long arms of chromosomes 6, 9, 11, 19 and 20, and, interestingly, DMs were found in two different size groups in a case of metastatic neuroblastoma (Franke, *et al.*, 1985).

If one takes deletion of certain genes on the short arm of one chromosome 1 as the crucial primary event for aggressive growth in neuroblastomas, our life table analysis can clearly distinguish two groups of patients – regardless of stage and age – one with a less than 10% survival chance *versus* the other with a 90% survival probability. This is superior to N-*myc* oncogene determinations in that 55 patients with only a single copy of the gene in their tumour DNA had an estimated survival rate of less than 60% (Seeger *et al.*, 1985).

Neuroblastoma cells with undisturbed homologues of chromosome 1 – probably a completely different entity – might just be temporarily deregulated or because of increased DNA content more vulnerable to chemotherapy.

We thank our colleagues from other German paediatric centers (Dortmund, Hannover, Neuss, Stuttgart, Tübingen, Berlin, Nürnberg, Bayreuth, Bielefeld, St Augustin, Heidelberg, Marburg, Kassel, Köln, Göttingen, Erlangen, Nordhorn, Würzburg, Herne) besides Gießen who supplied us with tumour material. This work was supported by the Deutsche Forschungsgemeinschaft (SFB 215) and the Parents' initiative Giessen. The technical help of Miss Rosel Engel, and the secretarial assistance of Mrs Marlies Mourek are gratefully appreciated.

### References

BERTHOLD, F., BRANDEIS, W.E. & LAMPERT, F. (1986). Neuroblastoma: Diagnostic advances and therapeutic results in 370 patients. *Monogr. Paediat.*, **18,** 206 (Karger, Basel).

BRODEUR, G.M., GREEN, A.A., HAYES, F.A., WILLIAMS, K.J., WILLIAMS, D.L. & TSIATIS, A.A. (1981). Cytogenetic features of human neuroblastomas and cell lines. *Cancer Res.*, **41,** 4678.

BRODEUR, G.M., SEEGER, R.C., SCHWAB, M., VARMUS, H.E. & BISHOP, J.M. (1984). Amplification of N-*myc* in untreated human neuroblastomas correlates with advanced disease stage. *Science*, **224,** 1121.

CHRISTIANSEN, H., FRANKE, F., BARTRAM, C.R. & 5 others (1987). Evolution of tumor cytogenetic aberrations and N-*myc* oncogene amplification in a case of disseminated neuroblastoma. *Cancer Genet. Cytogenet.*, **26,** 235.

EVANS, A.E., D'ANGIO, G.J., PROPERT, K., ANDERSON, J. & HANN, H.L. (1987). Prognostic factors in neuroblastoma. *Cancer*, **59,** 1853.

FRANKE, F., FÖRSTER, W., RUDOLPH, B. & LAMPERT, F. (1985). Metastatic neuroblastoma in an infant: Translocation (1;11), deletion (2) and double minute chromosomes. *Eur. J. Pediatr.*, **143,** 305.

FRANKE, F., RUDOLPH, B., CHRISTIANSEN, H., HARBOTT, J. & LAMPERT, F. (1986*a*). Tumour karyotype may be important in the prognosis of human neuroblastoma. *J. Cancer Res. Clin. Oncol.*, **111,** 266.

FRANKE, F., RUDOLPH, B. & LAMPERT, F. (1986*b*). Translocation (19;?) in two stage II neuroblastomas. *Cancer Genet. Cytogenet.*, **20,** 129.

GANSLER, T., CHATTEN, J., VARELLO, M., BUNIN, G.R. & ATKINSON, B. (1986). Flow cytometric DNA analysis of neuroblastoma. Correlation with histology and clinical outcome. *Cancer*, **58,** 2453.

GILBERT, F., FEDER, M., BALABAN, G. & 6 others (1984). Human neuroblastomas and abnormalities of chromosomes 1 and 17. *Cancer Res.* **44,** 5444.

HAYASHI, Y., HABU, Y., FUJII, Y., HANADA, R. & YAMAMOTO, K. (1986). Chromosome abnormalities in neuroblastomas found by VMA mass screening. *Cancer Genet. Cytogenet*, **22,** 363.

KANEKO, Y., KANDA, N., MASEKI, N. & 5 others (1987). Different karyotypic patterns in early and advanced stage neuroblastomas. *Cancer Res.*, **47,** 311.

LOOK, A.T., HAYES, F.A., NITSCHKE, R., MCWILLIAMS, N. & GREEN, A.A. (1984). Cellular DNA content as a predictor of response to chemotherapy in infants with unresectable neuroblastoma. *N. Engl. J. Med.*, **311,** 231.

ROSEN, N., REYNOLDS, C.P., THIELE, C.J., BIEDLER, J.L. & ISRAEL, M.A. (1986). Increased N-*myc* expression following progressive growth of human neuroblastoma. *Cancer Res.*, **46,** 4139.

SCHWAB, M., ALITALO, K., KLEMPNAUER, K.-H. & 6 others (1983). Amplified DNA with limited homology to *myc* cellular oncogene is shared by human neuroblastoma cell lines and a neuroblastoma tumour. *Nature*, **305,** 245.

SEEGER, R.C., BRODEUR, G.M., SATHER, H. & 4 others (1985). Association of multiple copies of the N-*myc* oncogene with rapid progression of neuroblastomas. *N. Engl. J. Med.*, **313,** 111.


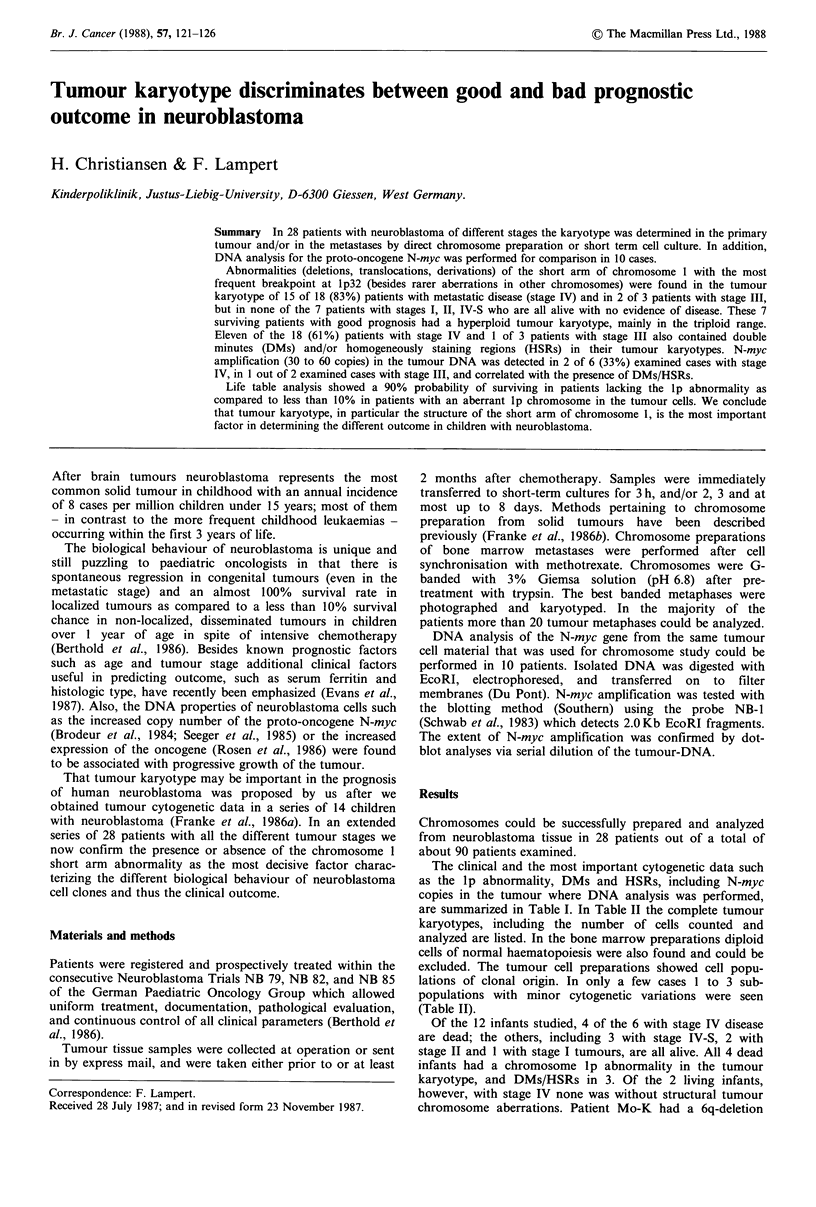

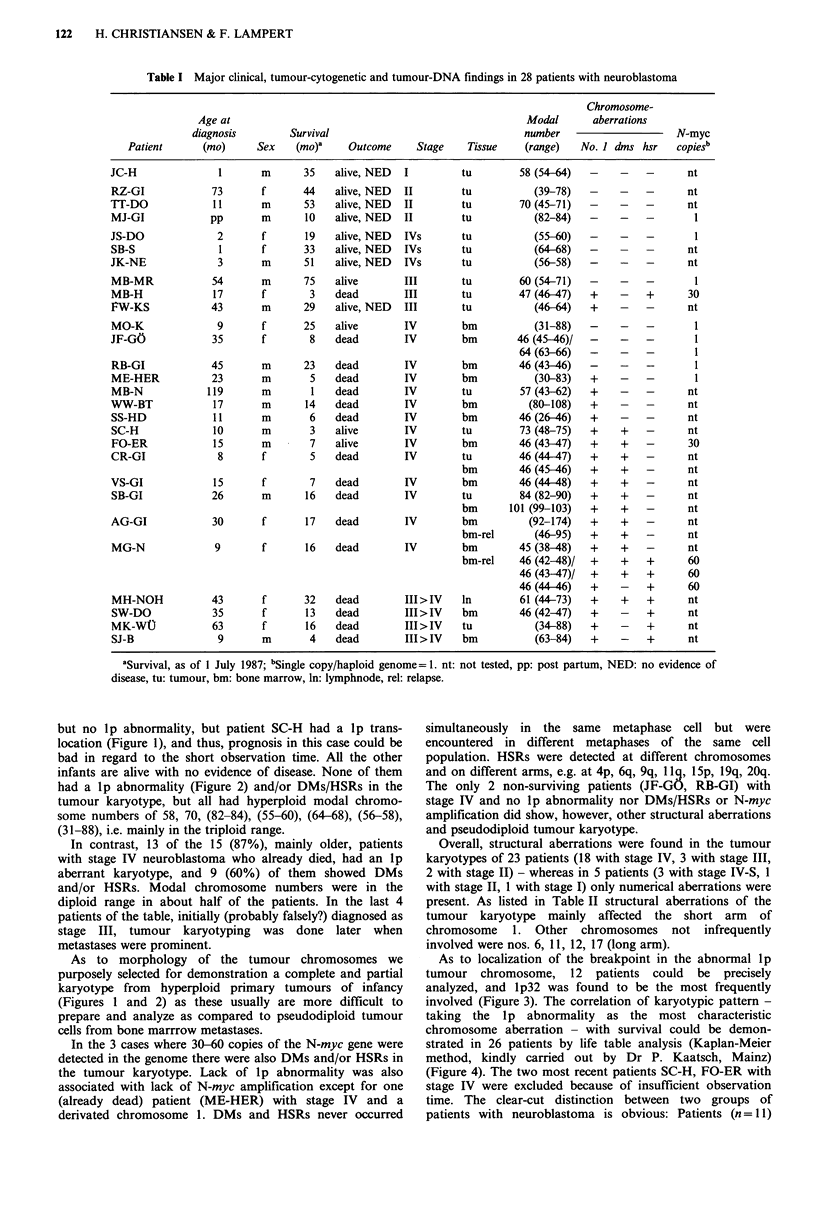

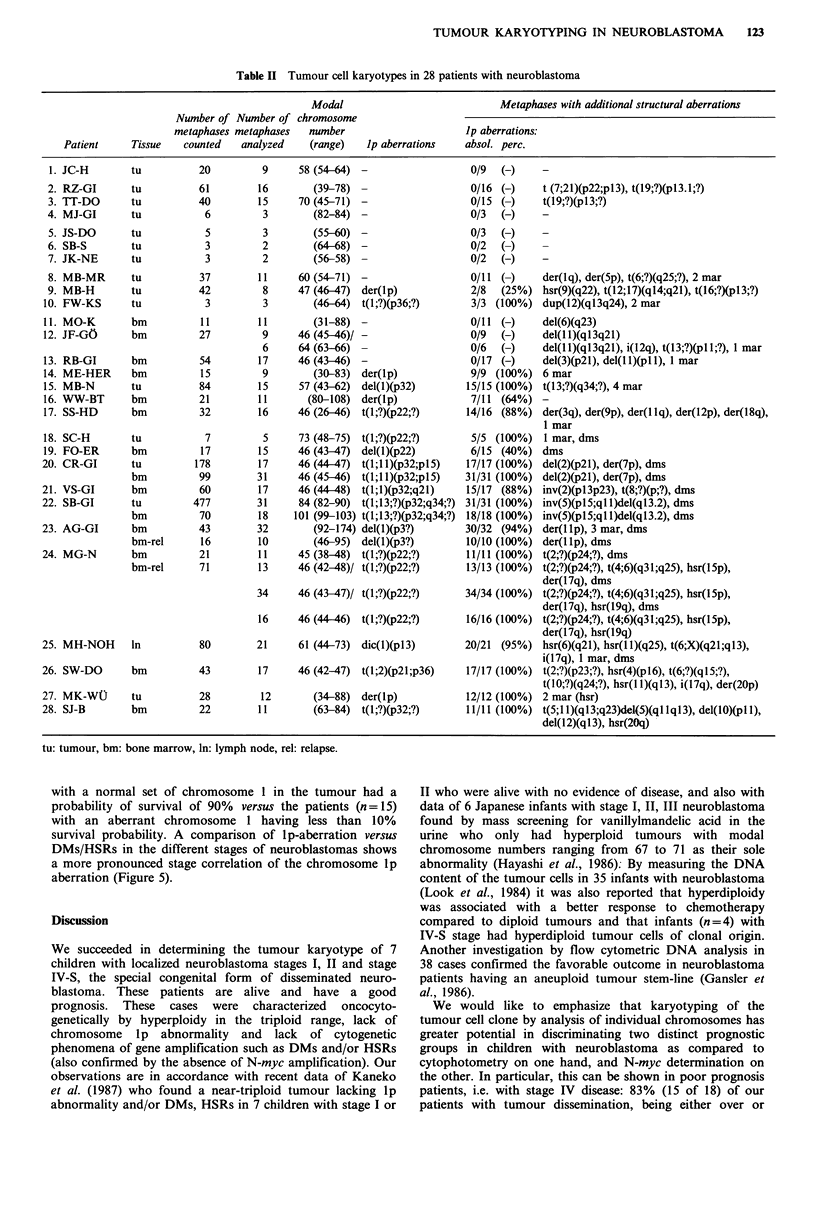

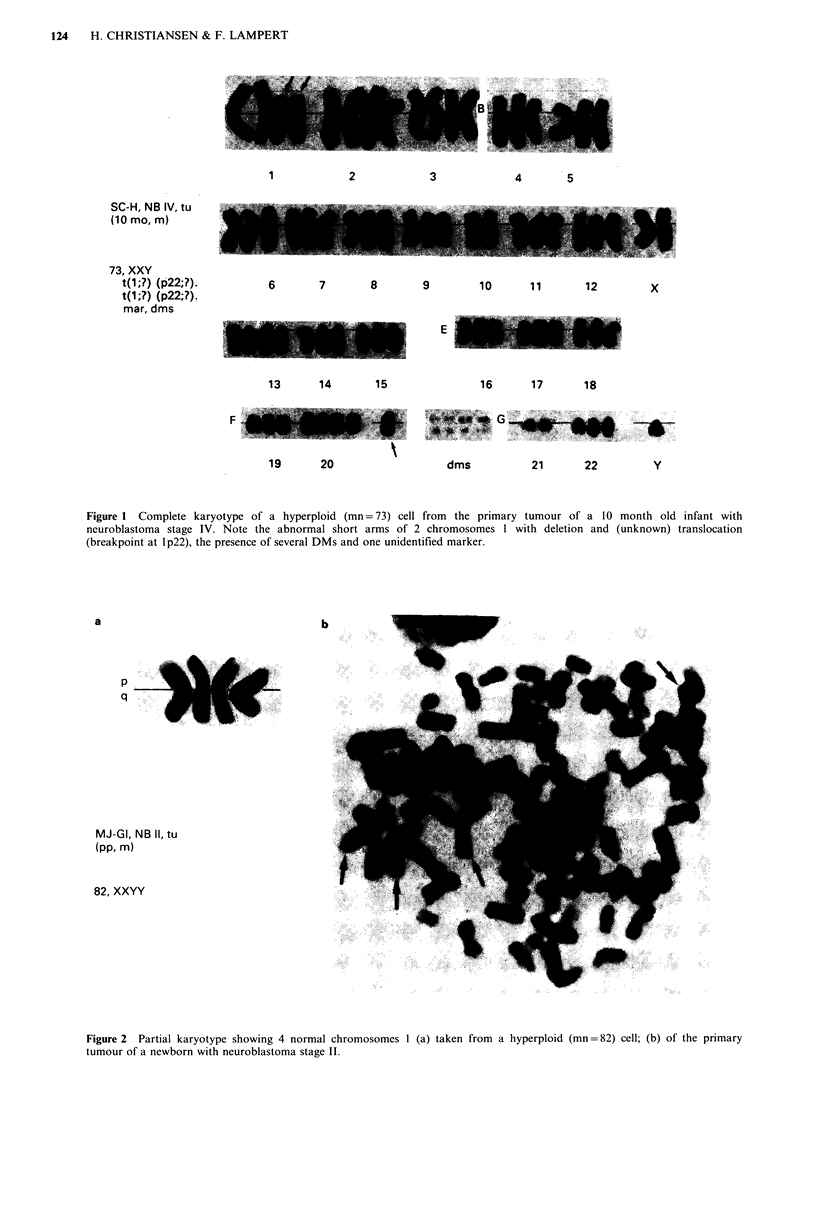

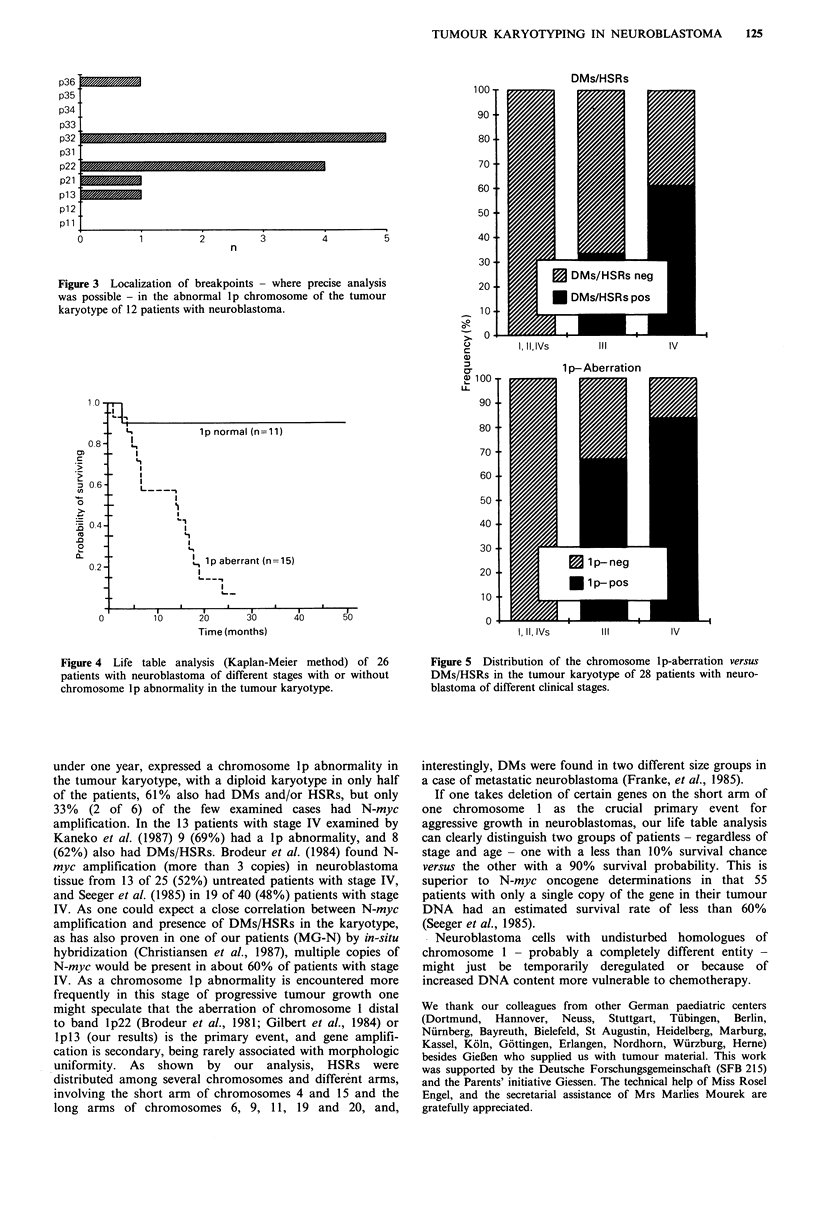

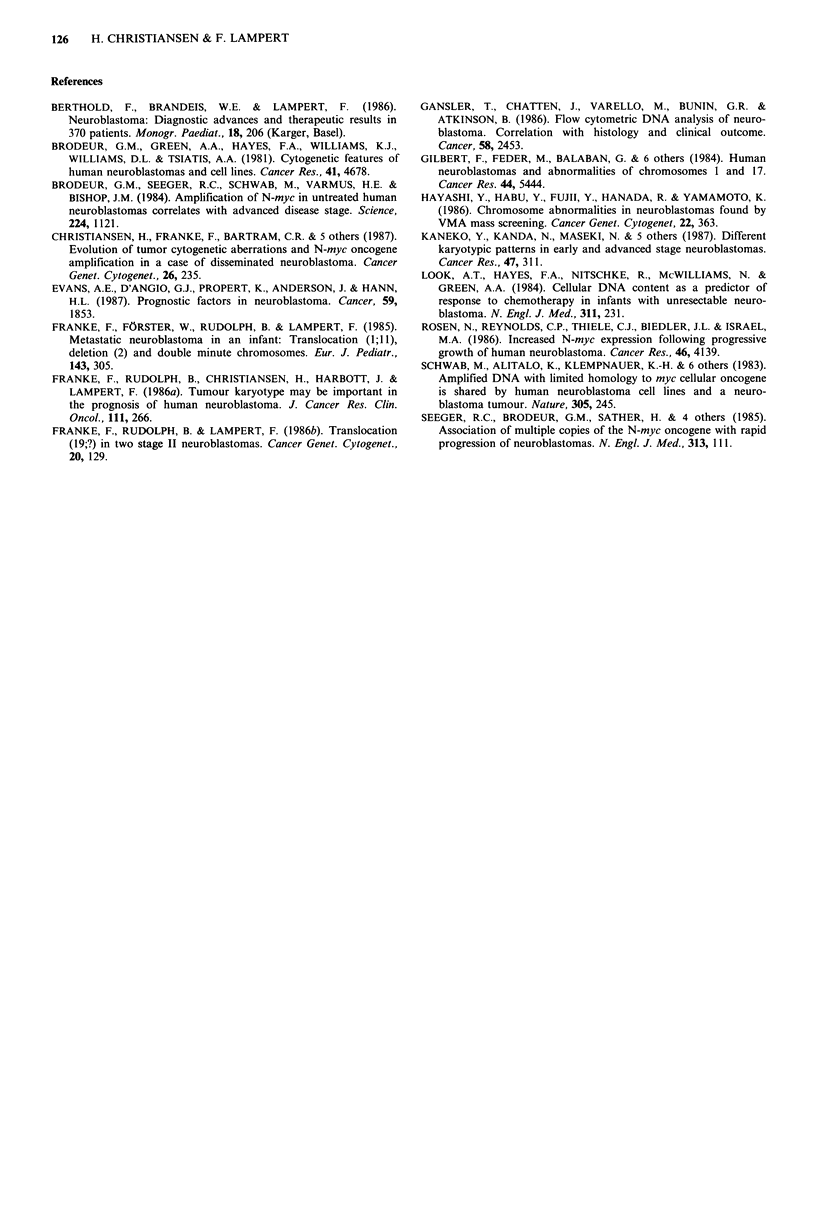


## References

[OCR_00841] Brodeur G. M., Green A. A., Hayes F. A., Williams K. J., Williams D. L., Tsiatis A. A. (1981). Cytogenetic features of human neuroblastomas and cell lines.. Cancer Res.

[OCR_00846] Brodeur G. M., Seeger R. C., Schwab M., Varmus H. E., Bishop J. M. (1984). Amplification of N-myc in untreated human neuroblastomas correlates with advanced disease stage.. Science.

[OCR_00852] Christiansen H., Franke F., Bartram C. R., Adolph S., Rudolph B., Harbott J., Reiter A., Lampert F. (1987). Evolution of tumor cytogenetic aberrations and N-myc oncogene amplification in a case of disseminated neuroblastoma.. Cancer Genet Cytogenet.

[OCR_00858] Evans A. E., D'Angio G. J., Propert K., Anderson J., Hann H. W. (1987). Prognostic factor in neuroblastoma.. Cancer.

[OCR_00863] Franke F., Förster W., Rudolph B., Lampert F. (1985). Metastatic neuroblastoma in an infant: translocation (1;11), deletion (2) and double minute chromosomes.. Eur J Pediatr.

[OCR_00869] Franke F., Rudolph B., Christiansen H., Harbott J., Lampert F. (1986). Tumour karyotype may be important in the prognosis of human neuroblastoma.. J Cancer Res Clin Oncol.

[OCR_00875] Franke F., Rudolph B., Lampert F. (1986). Translocation (19;?) in two stage II neuroblastomas.. Cancer Genet Cytogenet.

[OCR_00880] Gansler T., Chatten J., Varello M., Bunin G. R., Atkinson B. (1986). Flow cytometric DNA analysis of neuroblastoma. Correlation with histology and clinical outcome.. Cancer.

[OCR_00886] Gilbert F., Feder M., Balaban G., Brangman D., Lurie D. K., Podolsky R., Rinaldt V., Vinikoor N., Weisband J. (1984). Human neuroblastomas and abnormalities of chromosomes 1 and 17.. Cancer Res.

[OCR_00891] Hayashi Y., Habu Y., Fujii Y., Hanada R., Yamamoto K. (1986). Chromosome abnormalities in neuroblastomas found by VMA mass screening.. Cancer Genet Cytogenet.

[OCR_00901] Look A. T., Hayes F. A., Nitschke R., McWilliams N. B., Green A. A. (1984). Cellular DNA content as a predictor of response to chemotherapy in infants with unresectable neuroblastoma.. N Engl J Med.

[OCR_00907] Rosen N., Reynolds C. P., Thiele C. J., Biedler J. L., Israel M. A. (1986). Increased N-myc expression following progressive growth of human neuroblastoma.. Cancer Res.

[OCR_00912] Schwab M., Alitalo K., Klempnauer K. H., Varmus H. E., Bishop J. M., Gilbert F., Brodeur G., Goldstein M., Trent J. (1983). Amplified DNA with limited homology to myc cellular oncogene is shared by human neuroblastoma cell lines and a neuroblastoma tumour.. Nature.

